# Characterization of Extracellular Vesicles Derived From Human Precision‐Cut Liver Slices in Metabolic Dysfunction‐Associated Steatotic Liver Disease

**DOI:** 10.1002/jex2.70043

**Published:** 2025-04-30

**Authors:** Yana Geng, Ke Luo, Janine Stam, Dorenda Oosterhuis, Alan R. Gorter, Marius van den Heuvel, Rossella Crescitelli, Vincent E. de Meijer, Justina C. Wolters, Peter Olinga

**Affiliations:** ^1^ Department of Pharmaceutical Technology and Biopharmacy, Groningen Research Institute of Pharmacy University of Groningen Groningen the Netherlands; ^2^ Department of Analytical Biochemistry, Groningen Research Institute of Pharmacy University of Groningen Groningen the Netherlands; ^3^ Division of Pathology, Department of Pathology and Medical Biology University of Groningen, University Medical Center Groningen Groningen the Netherlands; ^4^ Department of Surgery, Sahlgrenska Center for Cancer Research and Wallenberg Centre for Molecular and Translational Medicine, Institute of Clinical Sciences Sahlgrenska Academy, University of Gothenburg Göteborg Sweden; ^5^ Department of Surgery, Section of Hepatobiliary Surgery & Liver Transplantation University of Groningen, University Medical Center Groningen Groningen the Netherlands; ^6^ Department of Pediatrics University Medical Center Groningen, University of Groningen Groningen the Netherlands

**Keywords:** biomarker, hepatic fibrosis, human precision‐cut liver slices, metabolic dysfunction‐associated steatohepatitis, proteomics

## Abstract

Extracellular vesicles (EVs) are cell‐produced, membrane‐surrounded vesicles that harbour the biological features of donor cells. In the current study, we are the first to isolate and characterize EVs isolated from human precision‐cut liver slices (PCLS), obtained from both healthy and metabolic dysfunction‐associated steatohepatitis (MASH) cirrhotic livers. PCLS derived from patients can faithfully represent disease conditions in humans. EVs were isolated from human PCLS after incubating in normal medium or modified medium that mimics the pathophysiological environment of metabolic dysfunction associated liver disease (MASLD). MASH PCLS produced higher amounts of EVs compared to healthy PCLS (*p* < 0.001). Mass spectrometry revealed that around 300 proteins were significantly different in EVs derived from MASH PCLS versus healthy PCLS (FDR < 0.05), irrespective of the type of medium. Significantly changed EV proteins were largely involved in signalling receptor binding function and showed potential in promoting fibrosis. In the liver, these ligand‐associated receptors are highly expressed in hepatic stellate cells, and the MASH EVs functionally promoted the activation of hepatic stellate cells. Furthermore, the amounts of EpCAM and ITGA3 in EVs were positively associated with the progression of MASLD, which suggests the use of liver‐derived EVs as potential biomarkers for MASLD. Characterization of EVs derived from human PCLS may assist future studies in investigating the pathogenesis and identifying liver‐specific EVs as biomarkers of MASLD.

**Abbreviations**
MASLD: metabolic dysfunction‐associated steatotic liver disease
MASH: metabolic dysfunction‐associated steatohepatitis
PCLS: precision‐cut liver slices
EVs: extracellular vesicles
CM: conditioned medium
ATP: adenosine triphosphate
TG: triglyceride
PDGFB: platelet‐derived growth factor subunit B
LTBP1: latent transforming growth factor β binding protein 1
TIMP3: metalloproteinase inhibitor 3
EpCAM: epithelial cell adhesion molecule
ITGA3: integrin subunit α3.

## 1 Introduction

Extracellular vesicles (EVs) are cell‐produced membrane‐surrounded vesicles. Their cargo reflects the biological features of the donor cells. However, as a major limitation so far, previous studies almost exclusively investigated EVs derived either from mono‐cultured cells (cell lines or primary cells), which poorly represent the pathogenic environment due to two‐dimensional (2D) culture, or from biofluids (e.g., serum) (Nakao et al. 2021; Povero et al. 2020; Thietart and Rautou 2020), which are difficult to identify the tissue of origin. To overcome these limitations, we isolated EVs from cultured human liver tissues, in the form of precision‐cut liver slices (PCLS). PCLS represents an ex vivo model based on liver tissue culture that preserves the multicellular architecture of the tissue and allows intercellular communication. In addition, PCLS prepared from human tissues of healthy or diseased livers offers an authentic representation of human physiological or pathological conditions.

Metabolic dysfunction‐associated steatotic liver disease(MASLD), previously known as non‐alcoholic fatty liver disease (NAFLD), has emerged as one of the major threats to public health, affecting more than 30% of the population worldwide (Arrese et al. 2021; Targher et al. 2021; Riazi et al. 2022). The development of MASLD comprises a spectrum of clinicopathological phenomena ranging from simple steatosis to metabolic dysfunction‐associated steatohepatitis (MASH) and could later progress to cirrhosis and hepatocellular carcinoma if no intervention is applied. Both clinical and experimental data indicated that hepatic fibrosis is an important threat factor and a primary predictor of mortality in MASH patients (Schwabe et al. 2020). Therefore, pinpointing the fibrotic stage is critical in creating therapeutic treatment plans in clinical practice. Besides the intra‐hepatic complications, MASLD is considered a multisystem disease that is commonly associated with comorbidity of cardiovascular disease, chronic kidney disease, and so forth (Targher et al. 2021; Byrne and Targher 2015). However, despite the increasing understanding of MASLD, there are still major gaps in our knowledge regarding the pathophysiology of the disease and the lack of reliable and reproducible biomarkers in the disease diagnosis and prognosis (Nassir 2022).

Increasing studies have shown that EVs reflect the pathological state of the liver. In MASLD, EVs derived from stressed hepatic cells of both parenchymal and non‐parenchymal cells harbour specific cargos including proteins (Guo et al. 2019; Hirsova et al. 2016), nucleic acids (Povero et al. 2015; Jiang et al. 2020), lipids (Dasgupta et al. 2020), and iron (Gao et al. 2022) that contribute to the progression of the disease. Moreover, the liver‐derived EVs were able to help predict the severity and mortality of liver diseases (Nakao et al. 2021; Povero et al. 2020; Thietart and Rautou 2020; Eguchi et al. 2022). Therefore, characterization of the EV cargo offers potential strategies in providing novel biomarkers, elucidating disease mechanisms, and serving as potential therapeutic tools in the treatment of MASLD.

Recently, we have developed a MASLD model by culturing liver slices in a modified medium, named GFIPO, which mimics the hyperglycaemia, hyperinsulinemia and hyperlipidaemia environment of MASLD (Prins et al. 2019). The previous and recent works from our group characterized the use of GFIPO for the establishment of MASLD in PCLS (Prins et al. 2019; Li et al. 2024). We have shown that GFIPO promotes the development of key MASLD features, including steatosis, inflammation and fibrosis in both mouse and human PCLS (Prins et al. 2019; Li et al. 2024). Additionally, long‐term incubation with GFIPO induces hepatocyte ballooning, a hallmark of MASLD progression (Li et al. 2024). Furthermore, it has been reported that removing a nutrient‐rich environment can reverse the MASLD phenotype (Ganguly et al. 2024; Aboujassoum et al. 2023; Reynes et al. 2014). Therefore, in the current study, we prepared human PCLS from both healthy livers (leftover tissue from partial hepatectomy or rejected donor livers) and MASH cirrhotic livers (liver explants from transplantations) and then incubated the PCLS in either standard medium or GFIPO medium to establish the early stage MASLD for healthy PCLS and to preserve the MASLD environment for MASH PCLS. After incubation, EVs were isolated from PCLS, which were subsequently characterized with a focus on EV proteins by untargeted proteomics. We aimed to study the differences between healthy EVs and MASH EVs, as well as the differences induced by the modified medium, to further explore their pathogenic roles and their potential usage as biomarkers in MASLD.

## 2 Materials and Method

### 2.1 Liver Tissues and Ethics Statement

**FIGURE 1:**
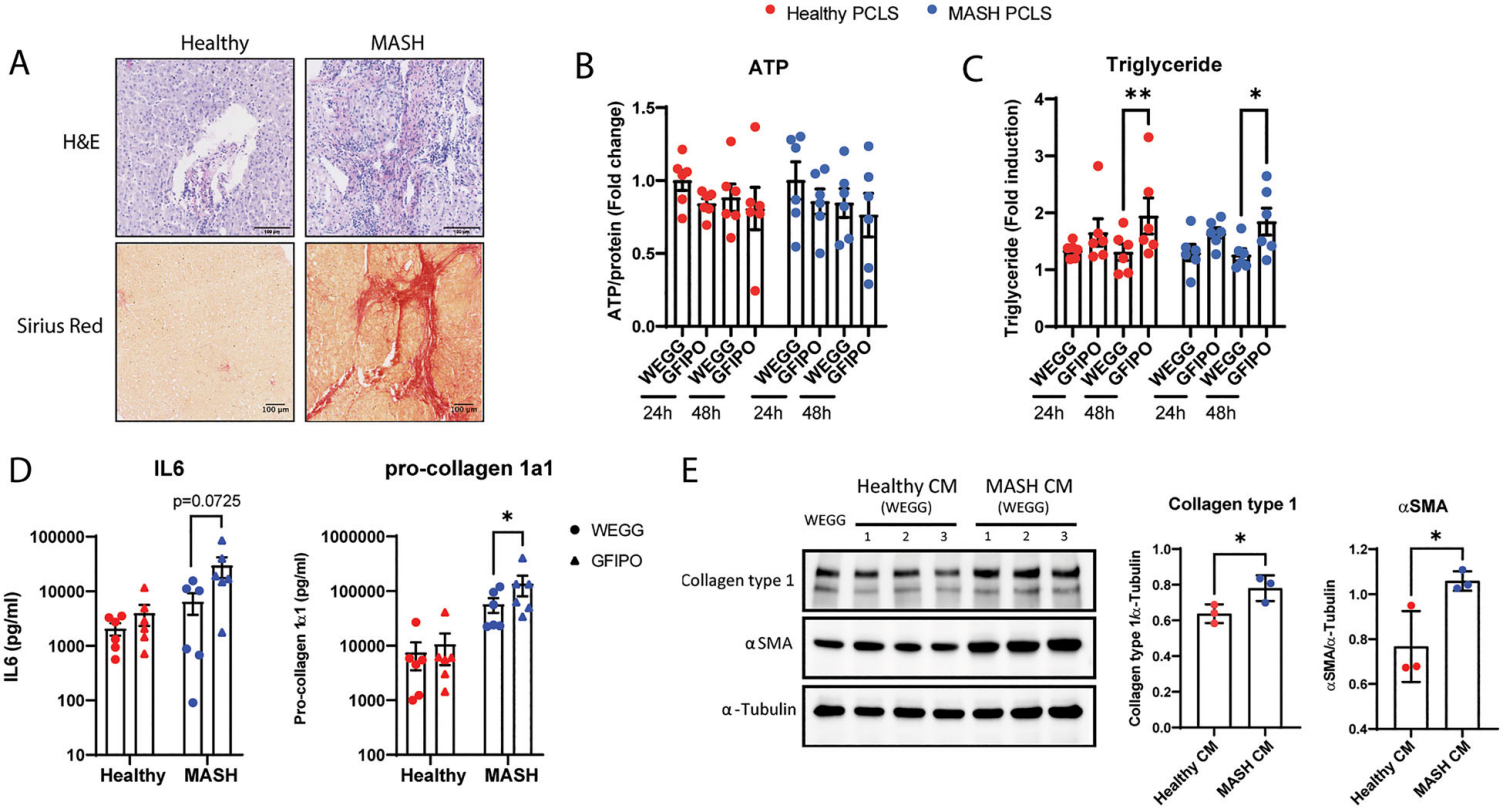
GFIPO promotes the MASLD phenotype in both healthy and MASH PCLS. (A) representative images of healthy and MASH livers in H&E staining and Sirius red staining. Scale bar = 100 µm. (B) ATP levels of PCLS after incubating for 24 and 48 h. (C) Triglyceride levels of PCLS after incubating for 24 and 48 h. (D) Pro‐collagen 1a1 and IL‐6 secretions from healthy and MASH PCLS. (E) LX2 cells were cultured in 25% (v/v) of conditioned medium (WEGG) of healthy or MASH PCLS for 48 h. Protein levels of collagen type 1, αSMA and α‐tubulin were measured in the cell lysates by western blot. ^*^Indicates *p* < 0.05, ^**^indicates *p* < 0.01. CM, conditioned medium; MASLD, metabolic associated steatotic liver disease.

Clinically healthy liver tissues were obtained from leftover material after partial hepatectomy or from donor livers unsuitable for transplantation. MASH cirrhotic liver tissues were obtained from leftover material during liver transplantation. MASH cirrhotic livers were referred to as MASH livers below. Liver tissues were stored in cold University of Wisconsin (UW) organ preservation solution until further use. The pathological characteristics of the livers were evaluated by histological scores on steatosis, hepatocyte ballooning, lobular inflammation and fibrosis based on the MASLD activity score (NAS) and fibrosis stage. Representative images of healthy and MASH livers are shown in Figure 1A. The H&E staining images of the livers used in this study are included in Figure S1. The scores were shown in Table S2. Livers with all scores of 0 were considered the ‘Healthy’ livers.

This study was approved by the Medical Ethical Committee of the University Medical Centre Groningen (UMCG). The use of human material was in accordance with Dutch legislation and the Code of Conduct for dealing responsibly with human tissue in the context of health research issued by the Committee on Regulation of Health Research, the COREON Foundation, refraining from the need for written consent for ‘further use’ of coded‐anonymous human tissue (https://www.coreon.org/wp‐content/uploads/2023/06/Code‐of‐Conduct‐for‐Health‐Research‐2022.pdf).

### 2.2 Preparation and Treatment of PCLS

PCLS were prepared according to the protocol described before (de Graaf et al. 2010), using Krumdieck tissue slicers. Slices were incubated in either a normal medium—Williams’ Medium E with GlutaMAX (Life Technologies, Carlsbad, California, USA) supplemented with 11 mmol/L glucose and 10 µg/mL gentamycin (WEGG) or a modified medium—Williams’ Medium E with GlutaMAX supplemented with 25 mmol/L glucose, 5 mmol/L fructose, 1 nmol/L insulin, 0.24 mmol/L palmitic acid, 0.48 mmol/L oleic acid and 10 µg/mL gentamycin (GFIPO) to mimic the MASLD pathophysiological environment (Prins et al. 2019). No serum was supplemented to either medium. Free fatty acids were conjugated to bovine serum albumin (BSA, Sigma‐Aldrich) prior to the supplementation. Slices were incubated at 37°C in an 80% O_2_/5% CO_2_ atmosphere while gently shaken. PCLS that were collected before incubation were marked as 0 h samples.

### 2.3 Cell Culture

LX2 cells (gift from Prof. Scott Friedman, Icahn School of Medicine at Mount Sinai) were maintained in Dulbecco's Modified Eagle Medium (DMEM, Invitrogen, Breda, the Netherlands) supplemented with 10% foetal calf serum (Invitrogen, Breda, the Netherlands), 100 U/mL penicillin and 100 µg/mL streptomycin (1% PS, Lonza, Verviers, Belgium) and cultured at 37°C in a 5% CO_2_ atmosphere. During the experiments, cells were incubated in the medium without foetal calf serum.

### 2.4 ATP Determination

The viability of the human PCLS was evaluated by measuring intracellular ATP and protein concentration as previously described (Suriguga et al. 2020). Briefly, ATP concentrations were measured using an ATP bioluminescence assay kit (Roche, Mannheim, Germany) in PCLS before and after incubation. The ATP level of each slice was normalized to its protein content. Protein contents were determined using a Lowry Protein Assay Kit (Bio‐Rad, Veenendaal, Netherlands). The slice with an ATP content above 2.0 nmol/mg of protein was considered viable based on previous studies that demonstrated a correlation between ATP content and liver slice viability, as assessed by morphological scoring (Westra et al. 2016). Moreover, PCLS did not survive the 24 h of incubation if the ATP level was lower than 2.0 nmol/mg protein.

### 2.5 Triglyceride Determination

Intracellular triglyceride (TG) was determined as previously described (Prins et al. 2019). Briefly, lipid was extracted from 3 slices and quantified using a Trig/GB kit (Roche Molecular Biochemicals, Almere, the Netherlands). The total lipid was normalized to its protein content.

### 2.6 Enzyme Linked Immunosorbent Assay

The secretions of pro‐collagen 1α1 and IL‐6 from PCLS were quantified by enzyme‐linked immunosorbent assay (ELISA). Briefly, the medium was collected and pooled from at least 6 slices and then stored at −80°C till measurement. Pro‐collagen 1a1 and IL‐6 were determined by the human pro‐collagen I alpha 1 ELISA Kit (Abcam, Cambridge, USA) and the human IL‐6 DuoSet ELISA (Bio‐Techne, Abingdon, UK), respectively, according to the manufacturers’ protocols. Optical densities were measured using a BioTek Synergy HT (BioTek Instruments), and wavelength correction was applied by subtracting readings at 540 nm from those at 450 nm.

### 2.7 RNA Isolation and Quantitative Polymerase Chain Reaction

The total RNA was extracted from the PCLS using a QIAzol lysis reagent (Molecular Research Center, Cincinnati, OH, USA) with a Qiagen RNeasy Lipid Tissue Mini kit (Qiagen, Venlo, the Netherlands) following the supplier's recommended protocol and as described before (Suriguga et al. 2022). RNA (1 µg) was converted into cDNA in 20 µL reaction mixtures using the Reverse Transcription System (Promega, Leiden, the Netherlands). Gene expressions were determined using the SYBR Green quantitative polymerase chain reaction protocol. The SYBR Green primers: EPCAM forward: 5’‐AATCGTCAATGCCAGTGTACTT‐3’; EPCAM reverse: 5’‐TCTCATCGCAGTCAGGATCATAA‐3’; ITGA3 forward: 5’‐TCCATCGGCAGACAGAGC‐3’; ITGA3 reverse: 5’‐GCACAGGTACACAGCACCAG‐3’; YWHAZ forward: 5’‐CCTGCATGAAGTCTGTAACTGAG‐3’; YWHAZ reverse: 5’‐GACCTACGGGCTCCTACAACA‐3’. The expressions of target genes were normalized to the expression of *YWHAZ*. The results were shown as 2^−ΔCt^.

### 2.8 Extracellular Vesicle Isolation

**FIGURE 2:**
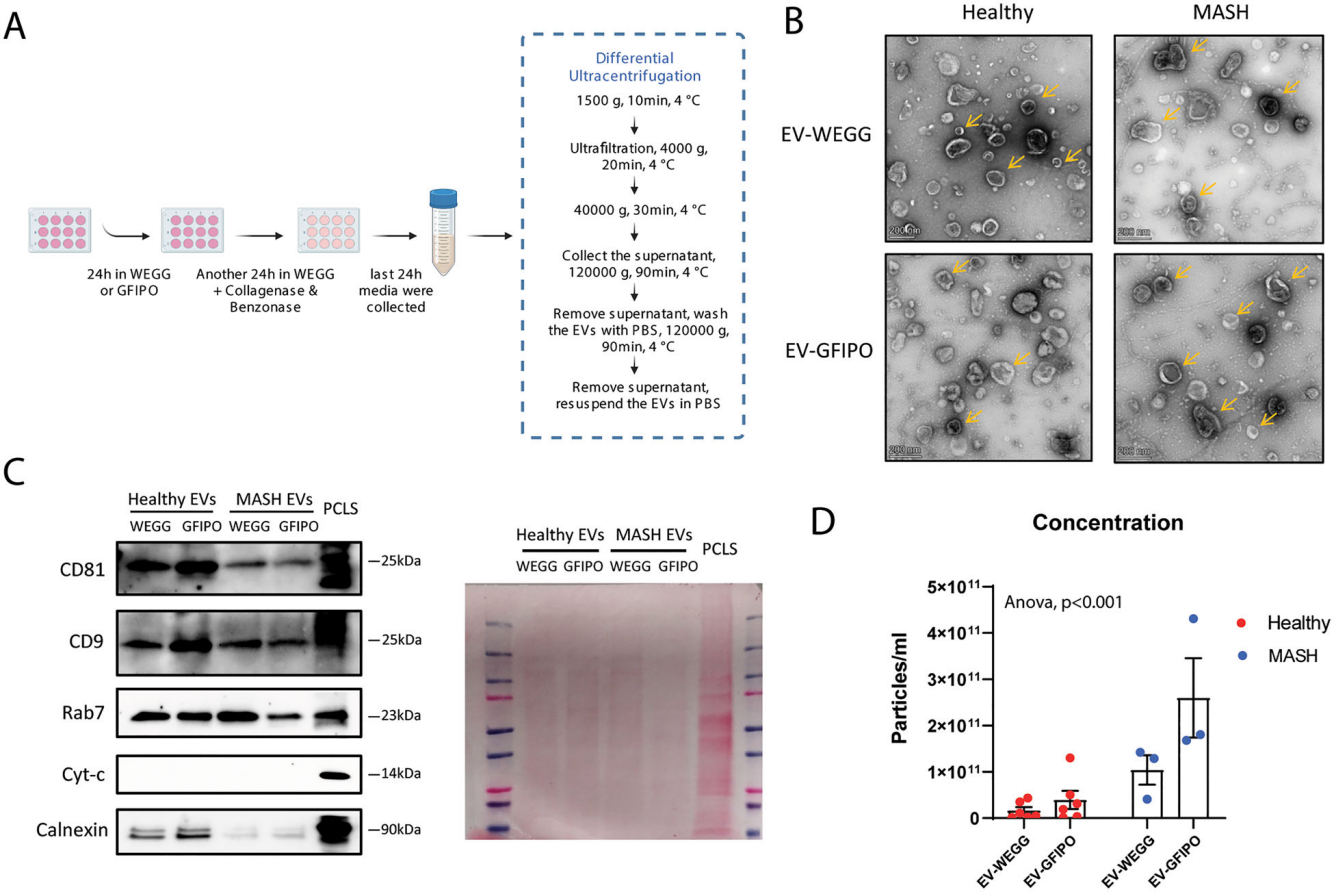
Isolation and characterization of extracellular vesicles (EVs) derived from human PCLS. (A) Workflow of EVs isolation. (B) Representative TEM pictures of EVs derived from healthy or MASH PCLS after incubating in WEGG or GFIPO. Yellow arrows indicate EVs. Scale bar = 200 nm. (C) CD81, CD9, Rab7, Cyt‐c and Calnexin were determined in EVs isolated from PCLS or PCLS lysate by western‐blot. A picture of Ponceau S staining shows the protein loading on SDS‐PAGE. (D) Concentrations of EVs derived from healthy (*n* = 6) or MASH (*n* = 3) PCLS that were incubated in WEGG or GFIPO were measured by nanoparticle tracking analysis (NTA). ^***^Indicates *p* < 0.001.

To isolate EVs, we used the protocol published by R. Crescitelli et al. (2021) with some modifications. PCLS were first incubated in either WEGG or GFIPO medium for 24 h, then transferred to WEGG medium containing collagenase D (2 mg/mL, Roche) and benzonase (50 U/mL, Sigma‐Aldrich) for another 24 h in order to release EVs from the extracellular matrix and prevent the aggregation of EVs. The supplementation of collagenase D and benzonase did not affect the viability and TG levels in both healthy and MASH PCLS (Figures S3A–S3C). EVs were isolated and enriched by differential ultracentrifugation as described before (Hernandez et al. 2020). Briefly, the conditioned medium (CM) was centrifuged at 1500 × *g* for 10 min at 4°C to remove possible cells. Supernatants were transferred into 100 kDa Amicon filter units (Sigma‐Aldrich) and centrifuged at 4000 × *g* for 20 min at 4°C to concentrate the CM. The concentrated CM was centrifuged at 40,000 × *g* for 30 min at 4°C to remove cell debris. Then the supernatant was further centrifuged at 120,000 × *g* for 90 min at 4°C. The EV pellet was suspended in cold PBS and centrifuged again at 120,000 × *g* for 90 min, and then re‐suspended in 150 µL PBS, followed by storage at −80°C for further use. A graphical illustration of the isolation procedure is depicted in Figure 2A.

### 2.9 Western Blot Analysis and Antibodies

The cell lysate was prepared for Western blot analysis as described previously (Geng et al. 2020). EV samples were added in RIPA buffer with or without 2‐mercaptoethanol (non‐reducing condition for CD9 and CD81) and then subjected to 4 freeze‐thaw cycles in liquid nitrogen before analysing the protein concentration. Protein concentrations were quantified using a Lowry protein assay kit. A total of 10 µg of proteins were loaded on Mini‐PROTEAN TGX precast 4%–15% gradient gels (Bio‐Rad) and transferred to 0.45 µm nitrocellulose membranes using the Trans‐Blot Turbo transfer system (Bio‐Rad). Membranes were blocked in 0.5% BSA and then incubated overnight in primary antibodies at 4°C. Primary antibody details and dilutions are listed in Table S1, and appropriate horseradish peroxidase (HRP)‐conjugated secondary antibodies (1:2000; DAKO, Agilent, Santa Clara, CA, USA) were used for detection. Proteins were detected using the Pierce ECL Western blotting kit (Thermo Fisher Scientific). Images were captured using the ChemiDoc XRS system and Image Lab version 3.0 (Bio‐Rad). Bands were quantified by determining the densitometry with ImageJ software (public domain, developed at the National Institutes of Health).

### 2.10 Nanoparticle Tracking Analysis (NTA)

The concentration of particles was estimated using the Nanosight NS300 unit (Malvern, Egham, UK). All samples were diluted to provide a concentration of 1 × 10^7^–1 × 10^8^ particles/mL counts. All counts were performed in replicates of 5 for each sample, collecting 60‐s videos with 300 valid tracks recorded per video (minimum of 1000 valid tracks recorded per sample). Nanosight 3.0 software was used for all analyses, using standard settings. To minimize variability and achieve optimal visualization of particles, the camera level was set at 13 with screen gain at 8.0 in light scatter mode, and the detection threshold was set at 2 for maximum sensitivity with a minimum of background noise for all experiments.

### 2.11 Transmission Electron Microscopy

Investigation of EVs by negative staining was performed as previously described (Crescitelli et al. 2021). Briefly, 3 µg of EVs was placed onto glow‐discharged 200‐mesh formvar/carbon copper grids (Electron Microscopy Sciences, Hatfield Township, PA). After two washes in H_2_O, EVs were fixed in 2.5% glutaraldehyde. After two further washes in H_2_O, the samples were stained with 2% uranyl acetate for 1.5 min. Negative‐stained samples were examined on a digitized Talos L120C electron microscope (Thermo Fisher Scientific) at 120 kV with a CCD camera.

### 2.12 Sample Preparation for Discovery‐Based Proteomics Analyses and Data Pre‐Processing

Protein levels were determined with discovery‐based proteomics (using label‐free quantification) for relative protein concentrations (Lin et al. 2023). Briefly, in‐gel digestion was performed on 30 µL of the enriched EVs using trypsin (150 ng sequencing grade modified trypsin V5111; Promega) after reduction with 10 mmol/L dithiothreitol and alkylation with 55 mmol/L iodoacetamide proteins (Wolters et al. 2016).

Discovery mass spectrometric analyses were performed on a quadrupole orbitrap mass spectrometer equipped with a nano‐electrospray ion source (Orbitrap Exploris 480, Thermo Scientific). Chromatographic separation of the peptides was performed by liquid chromatography (LC) on an Evosep system (Evosep One, Evosep) using a nano‐LC column (EV1137 Performance column 15 cm × 150 µm, 1.5 µm, Evosep; buffer A: 0.1% v/v formic acid, dissolved in milliQ‐H_2_O, buffer B: 0.1% v/v formic acid, dissolved in acetonitrile). Peptides were separated using the 30SPD workflow (Evosep). The mass spectrometer was operated in positive ion mode and data‐independent acquisition mode (DIA) using isolation windows of 16 m/z with a precursor mass range of 400–1000, switching the FAIMS between CV −45 V and −60 V, with three scheduled MS1 scans during each screening of the precursor mass range. LC‐MS raw data were processed with Spectronaut (version 16.0.220606) (Biognosys) using the standard settings of the directDIA workflow except that quantification was performed on MS1, with a human SwissProt database (www.uniprot.org, 20.350 entries). For the quantification, local normalization was applied, and the *Q* value filtering was set to the classic setting without imputing on. The MS‐based proteomics data used in this study is available upon request from the corresponding author.

### 2.13 Downstream Proteomic Data Processing

All data were analysed using Microsoft Excel and R statistical software. Raw intensities were log2 transformed. Proteins with missing values were removed in subsequent analyses. Vesiclepedia (Version 4.1, 2018) and Exocarta (July 2015) were downloaded to map previously reported EV proteins. Differential expression analysis was performed using a limma‐based R package, DEqMS, which was developed for proteomic analysis with low‐replicate samples (Ritchie et al. 2015). Gene ontology (GO) and KEGG pathway analyses were performed using g:Profiler. Ligand‐receptor interaction analysis was obtained using experimentally verified cognate binding partners based on Cellinker that catalogues literature‐supported ligand‐receptor interactions. The expression levels of ligand‐receptor bind partners in different human tissues were obtained from The Human Protein Atlas (http://www.proteinatlas.org). The Benjamini–Hochberg method was performed, and statistical significance is defined as FDR < 0.05.

### 2.14 Statistical Analysis

Data are shown as mean ± SEM (for PCLS and EVs results) or mean ± SD (for LX2 results). Differences between groups were compared by paired two‐tailed Student's *t*‐test or two‐way ANOVA test followed by Tukey's multiple comparisons test. All statistical analyses were performed with GraphPad Prism 5.0 (La Jolla, CA, USA) or with R v3.0.2 (www.r‐project.org). Differences were considered to be statistically significant when *p* < 0.05 or FDR < 0.05.

## 3 Results

### 3.1 GFIPO Promotes the MASLD Phenotype in Both Healthy and MASH PCLS

We have recently established a MASLD model in PCLS by culturing liver slices in a modified medium containing pathophysiological amounts of glucose (36 mmol/L), fructose (5 mmol/L), insulin (1 nmol/L), palmitic acid (240 µmol/L) and oleic acid (480 µmol/L) (GFIPO) (Prins et al. 2019). Human PCLS prepared from healthy (*n* = 6) or MASH (*n* = 6) livers were incubated in either a standard medium (WEGG, the formula is shown in ‘Material and Methods’) or the modified medium (GFIPO) for up to 48 h to develop or preserve MASLD phenotype, respectively. After incubation, PCLS remained alive and GFIPO did not affect the intracellular ATP levels in both healthy and MASH PCLS (*p* > 0.05, Figure 1B). Importantly, GFIPO significantly increased the intracellular TG content in both healthy and MASH PCLS at 48 h (54.68% and 54.33%, respectively, Figure 1C). Moreover, GFIPO increased the secretions of pro‐collagen 1α1 (41.35% and 139.58% in healthy and MASH PCLS, respectively) and IL‐6 (91.79 % and 363.17 % in healthy and MASH PCLS respectively), when compared to WEGG in both healthy and MASH PCLS (Figure 1D).

Because fibrogenesis is a crucial step in the progression of MASLD, we investigated whether PCLS secreted factors that modulate fibrosis by incubating hepatic stellate cells (LX2 cells) in the conditioned medium (CM) of PCLS. LX2 cells, although pre‐activated, remain a widely used model in HSC research, particularly because they can be further activated with various biological molecules (Xu et al. 2005). Firstly, we confirmed that neither the slice medium nor CM affects the viability of LX2 cells, as there were no significant differences in intracellular ATP levels in LX2 cells cultured in slice medium (WEGG and GFIPO) (Figure S2A) or CM (Figure S2B). Furthermore, the CM of MASH PCLS promoted the activation of LX2 cells compared to the CM of healthy PCLS, as demonstrated by the significantly increased intracellular collagen type 1 (22.47%) and αSMA (38.05%) in LX2 cells (*p* < 0.05, Figure 1E), which indicates secretion of pro‐fibrotic factors from MASH PCLS.

### 3.2 Isolation and Characterization of EVs Derived From Human PCLS

Next, we isolated EVs from the CM of PCLS that were collected from the last 24 h of incubation as depicted in Figure 2A. As shown by transmission electron microscopy (TEM) in Figure 2B, all four groups of EVs exhibit typical EV size and shape (round elements with sizes between 50 and 250 nm) and the pellet is composed of both small and large EVs. Western blot results showed that the isolated EVs were enriched with EV protein markers (including tetraspanins—CD81, CD9; Rab family protein: Rab7); meanwhile, the mitochondrial protein—cytochrome c (Cyt‐c) is absent in all EV groups (Figure 2C). We also observed a presence of calnexin in our EVs, which is probably due to contamination, though the signal is much stronger in PCLS (Figure 2C). Moreover, we estimated the amount of EVs by NTA. As shown in Figure 2D, MASH PCLS produced a significantly higher amount of EVs compared to healthy PCLS (*p* < 0.001, Figure 2D).

### 3.3 Proteomic Profiling of PCLS‐Derived EVs

**FIGURE 3:**
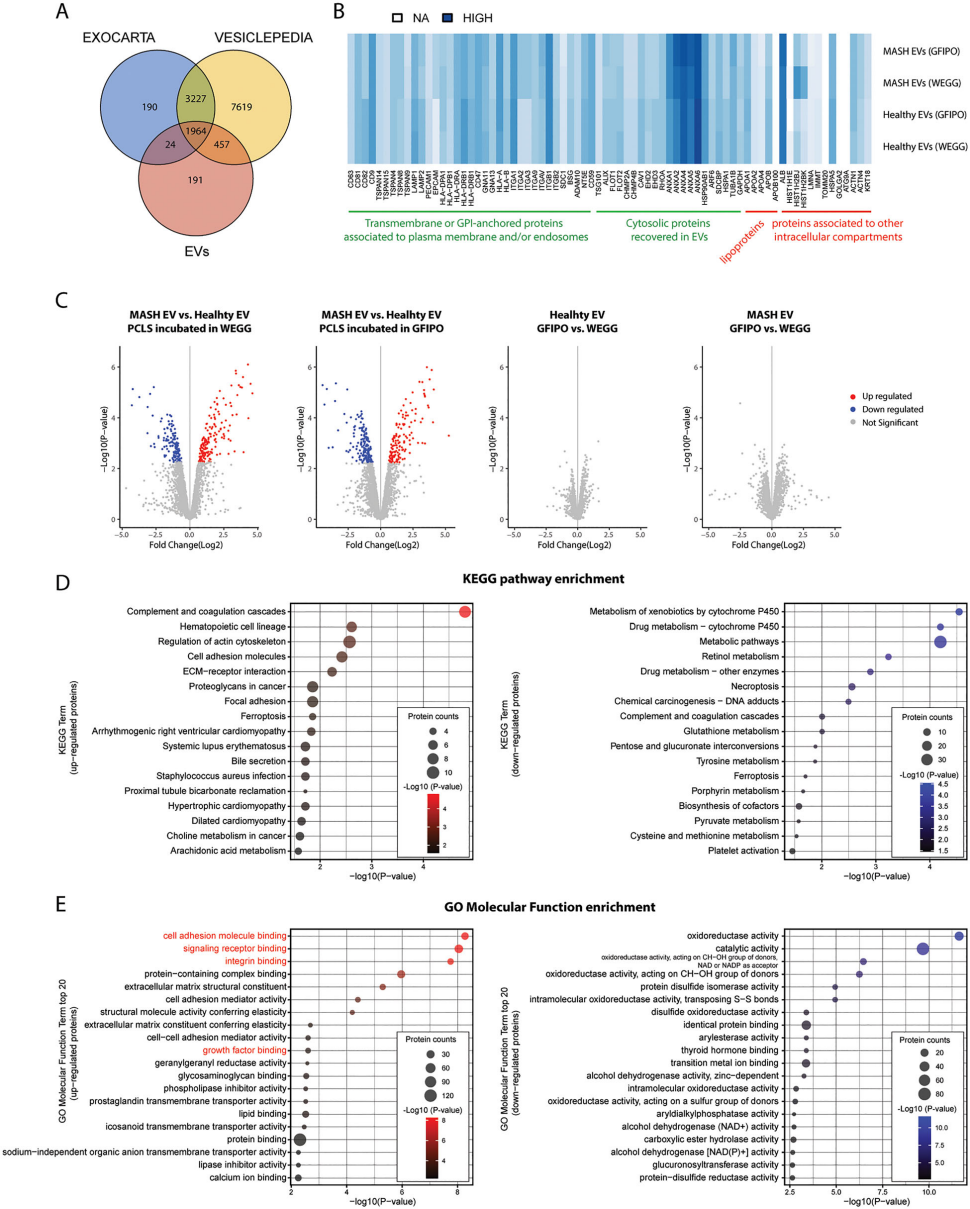
Proteome profiling of EVs. (A) Venn diagram of the proteins identified in all EVs compared to the proteins reported in Exocarta or Vesiclepedia. (B) Heatmap of both positive and negative EV markers in all groups of EVs. (C) Volcano plots of the differentially abundant proteins in each of the two EV groups. (D) KEGG pathway enrichment analysis of the up‐ or down‐regulated EV proteins in MASH (WEGG) EVs versus healthy (WEGG) EVs. (E) GO molecular function enrichment analysis of the significant up‐ or down‐regulated proteins in MASH (WEGG) EVs versus healthy (WEGG) EVs. MASH, metabolic dysfunction‐associated steatohepatitis.

To further characterize EVs, we performed untargeted proteomics on EVs isolated from healthy (*n* = 3) and MASH (*n* = 3) PCLS. In total, 4348 proteins were identified. We proceeded with the analysis with 2636 proteins that were present in all EV samples, of which 75% were catalogued in Exocarta and 92% were catalogued in Vesiclepedia (Figure 3A). Moreover, we assessed the enrichment of both positive and negative EV protein markers as stated in the MISEV2018 guidelines in our EV samples (Thery et al. 2018). Our EVs were highly enriched with the positive EV markers belonging to ‘transmembrane or GPI‐anchored proteins associated to plasma membrane and/or endosomes’ and ‘cytosolic proteins recovered in EVs.’ Although there was also some contamination of lipoproteins, albumin and proteins associated with other intracellular compartments (Figure 3B).

Next, we performed the differential analysis on each of the two EV groups. As shown in Figure 3C, MASH EVs showed great differences from healthy EVs, with 293 significantly different proteins when PCLS were incubated in WEGG and 316 significantly different proteins when PCLS were incubated in GFIPO (FDR < 0.05). The results of differential protein expression of MASH EVs (WEGG) versus healthy EVs (WEGG) are listed in Table S3. However, when comparing GFIPO to WEGG, there were no significant changes in protein levels in either healthy or MASH EVs (FDR > 0.05, Figure 3C). Figure S4A presents the top 100 significantly different proteins, which exhibit distinct patterns between the healthy EVs and MASH EVs. To obtain functional indications of the enriched EV proteins, we performed KEGG pathway enrichment and GO molecular function (GOMF) enrichment analysis on the significantly up‐regulated or down‐regulated proteins in MASH (WEGG) EVs versus healthy (WEGG) EVs. The up‐regulated proteins are highly enriched in ‘complement and coagulation cascades,’ while the down‐regulated proteins are largely enriched in several KEGG terms related to xenobiotics or drug metabolisms (Figure 3D). With regard to GOMF, the up‐regulated proteins are highly enriched for molecule/receptor binding functions, for example, ‘cell adhesion molecule binding’ and ‘signaling receptor binding,’ meanwhile, the down‐regulated proteins are largely enriched in ‘oxidoreductase activity’ and ‘catalytic activity’ (Figure 3E). The different proteins of MASH (GFIPO) EVs versus healthy (GFIPO) EVs showed similar enrichment results, which are shown in Figure S4C. Given that EVs are important messengers in signalling activation, we next looked into the ligand‐receptor interactions of the enriched EV proteins.

### 3.4 Ligand‐Receptor Interaction Analysis of Differentially Regulated Proteins in MASH EVs

**FIGURE 4:**
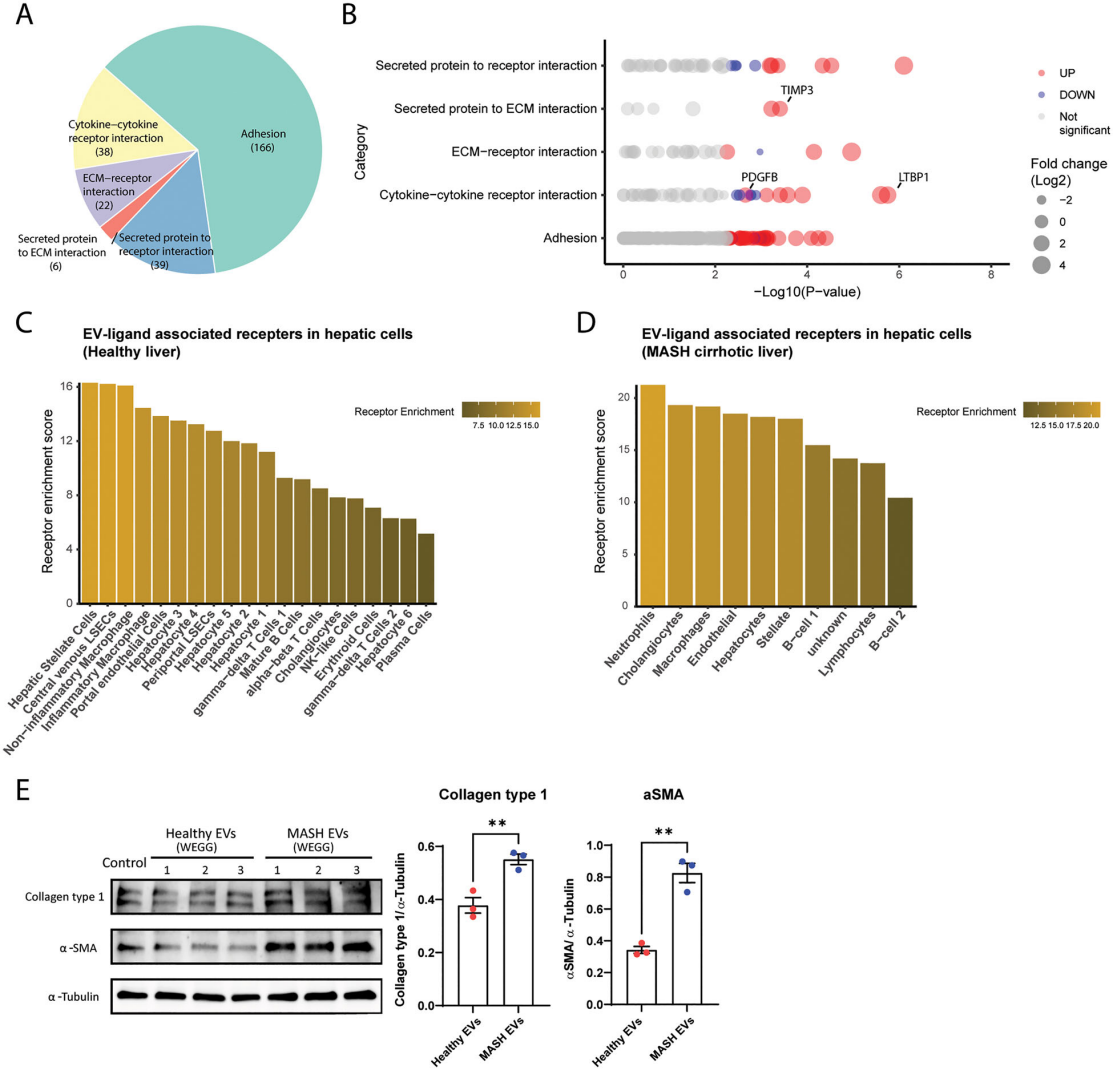
Ligand‐receptor interactions of the EV proteins. (A) The enriched ligands in EVs were subjected to ligand‐receptor interaction analysis using the manually curated database Cellinker. Pie chart of the distribution of the interactions of enriched EV ligands. (B) Significantly changed ligands are highlighted in every interaction type. (C) Bar plots represent the collective expressions of EV protein‐associated receptors in each type of hepatic cell using the single‐cell RNA‐seq data of healthy individuals from S. A. MacParland et al. (2018) (D) Bar plots represent the collective expressions of EV protein‐associated receptors in each type of hepatic cell using the single nucleus RNA‐seq data of the end‐stage MASH cirrhotic patients (Gribben et al. 2024). (E) LX2 cells were incubated with healthy EVs or MASH EVs (10 µg/mL) for 48 h. The protein levels of collagen type 1 and αSMA were measured in the cell lysate. ^**^Indicates *p* < 0.01.

To gain insight into these interactions of the enriched proteins in MASH EVs, we used the experimentally verified ligand‐receptor database Cellinker, which catalogued and categorized the ligand‐receptor interactions. By employing the EV proteins as ligands, we identified 271 ligand‐receptor binding partners, encompassing 166 adhesion interactions, 38 cytokine‐cytokine receptor interactions, 22 ECM‐receptor interactions, 6 secreted proteins to ECM interactions and 39 secreted proteins to receptor interactions (Figures 4A and B). A list of the EV ligands and their associated receptors can be found in Table S4. To assess the potential target organs and target cells of MASH EVs, we calculated the abundance of the EV protein‐associated receptors across all tissues based on their expressions provided by The Human Protein ATLAS (https://www.proteinatlas.org/). The significantly upregulated receptors are highly expressed in several organs, including the liver (Figure S4E). To further estimate the potential functions of the MASH EVs in the liver, we calculated the expressions of these EV‐ligand‐associated receptors across all types of hepatic cells, using the human liver single‐cell RNA‐seq data from S. A. MacParland et al. (2018). This liver transcriptomic profile was created from 5 healthy donor livers and reported 20 distinct hepatic cell populations, including both parenchymal and non‐parenchymal cells (MacParland et al. 2018). Among all the 20 hepatic cell clusters, hepatic stellate cells demonstrated the highest expression of EV‐ligand‐associated receptors, followed by central venous liver sinusoidal endothelial cells and macrophages (Figure 4C). Moreover, to investigate the potential acceptor cells in MASH liver, we utilized a recent snRNA‐seq data from MASH cirrhotic patients (Gribben et al. 2024). In contrast to healthy livers, in MASH cirrhotic livers, neutrophils exhibit the highest enrichment score for EV receptors, followed by cholangiocytes, macrophages, endothelial cells, hepatocytes, and hepatic stellate cells (Figure 4D). Notably, the receptor enrichment scores for cholangiocytes, macrophages, endothelial cells, hepatocytes, and hepatic stellate cells were relatively similar. Based on these findings, hepatic stellate cells could be one of the main acceptors for EVs in both healthy and MASH cirrhotic livers. Importantly, to functionally verify the actions of MASH EVs on hepatic stellate cells, we incubated LX2 cells with healthy EVs or MASH EVs (10 µg/mL). After 48 h of incubation, MASH EVs significantly increased collagen type 1 and αSMA in LX2 cells compared to healthy EVs (*p* < 0.01, Figure 4E), which indicates that MASH EVs contain fibrogenic factors that could promote the activation of hepatic stellate cells. Several significantly upregulated ligands in MASH EVs could explain their hepatic stellate cell‐activating roles, for example, platelet‐derived growth factor subunit B (PDGFB), latent transforming growth factor β binding protein 1 (LTBP1) and metalloproteinase inhibitor 3 (TIMP3) (Figure 4B), which are known to activate hepatic stellate cells and promote fibrosis (Schwabe et al. 2020; Rifkin et al. 2022).

### 3.5 Hepatic EV‐Enriched EpCAM and ITGA3 as Potential Biomarkers for MASLD

**FIGURE 5:**
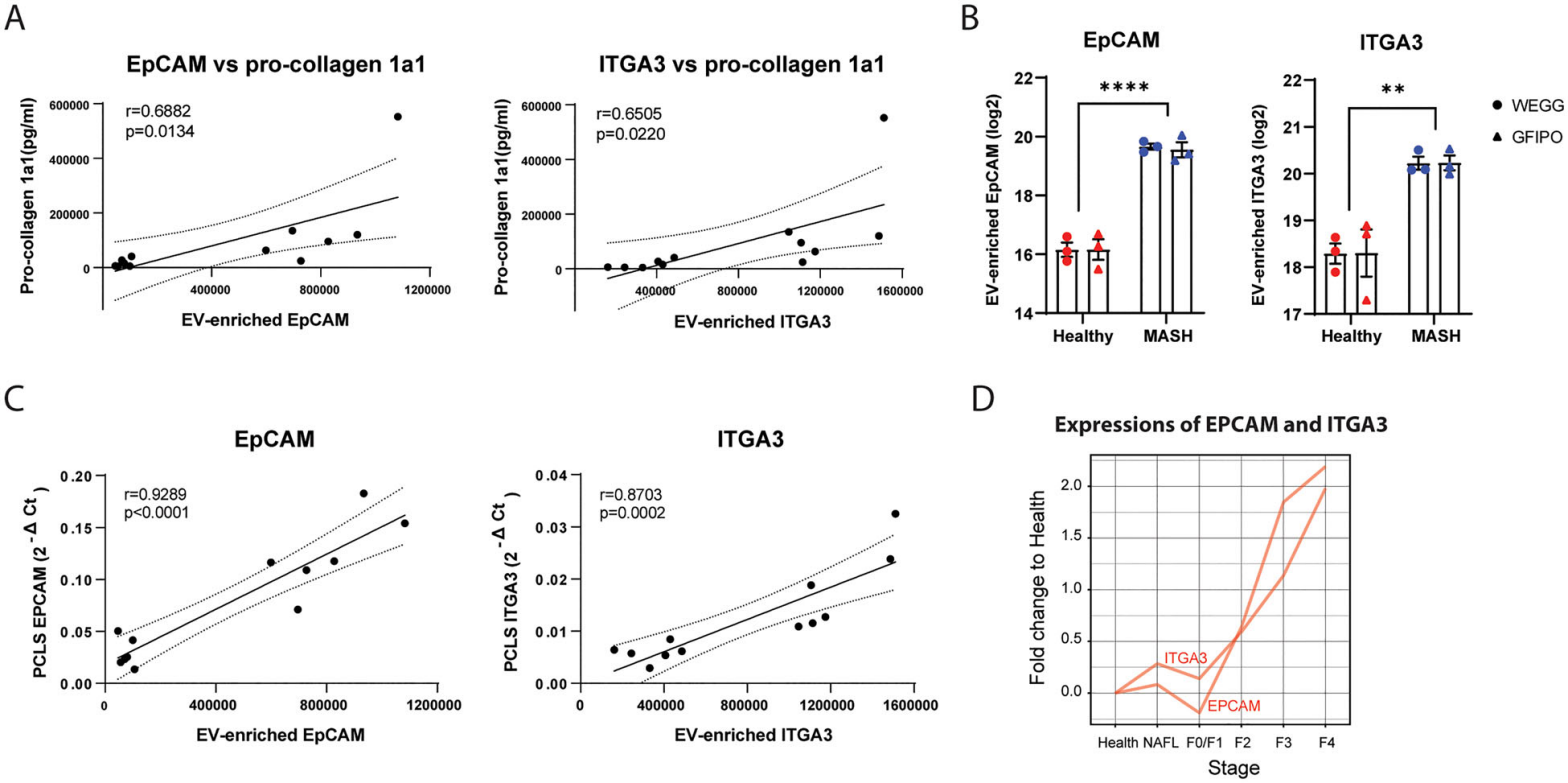
Hepatic EV‐enriched EpCAM and ITGA3 as potential biomarkers for MASLD. (A) EV‐enriched EpCAM and ITGA3 are positively correlated to the levels of pro‐collagen 1a1 secreted from PCLS, which were measured at 48 h. (B) the protein levels of EpCAM and ITGA3 in healthy and MASH EVs after incubation in WEGG or GFIPO. (C) EV‐enriched EpCAM and ITGA3 are positively correlated to their hepatic mRNA expressions. (D) the expressions of EPCAM and ITGA3 across MASLD stages, which were published by O. Govaere et al. (2020). ^**^Indicates *p* < 0.01. ^****^Indicates *p* < 0.0001.

EVs have shown great potential as diagnostic and prognostic biomarkers in clinical use. Therefore, we evaluated our EV proteins with regard to their use as potential biomarkers in MASLD. Since hepatic fibrosis is the most important indicator and predictor of the morbidity and mortality of MASLD (Schwabe et al. 2020), we looked into the EV proteins, which were positively associated with hepatic fibrosis. Specifically, 11 EV proteins showed a significant correlation with the secretion of pro‐collagen 1α1 from the corresponding PCLS (*p* < 0.05, Table S5). Among them, 4 proteins (EPCAM, ITGA3, SDCBP2 and CLDN4) showed significant and continuous increase throughout the progression of MASLD, particularly upon the transition from NAFL to NASH (Figure S5A). Notably, EPCAM and ITGA3 demonstrated less variation between individuals within each group (Figure S5B), which further supports their potential as reliable biomarkers. Therefore, EPCAM and ITGA3 were chosen for further analysis. An overview table with correlation scores for all proteins is shown in Table S5. As shown in Figure 5A, EpCAM and ITGA3 were significantly and positively correlated to the secretion of pro‐collagen 1a1 (*r* = 0.6882 and *r* = 0.6505, respectively, *p* < 0.05, Figure 5A). Moreover, EpCAM and ITGA3 were significantly increased in all MASH EVs compared to healthy EVs (*p* < 0.0001 and *p* < 0.01, respectively, Figure 5B). Since the protein levels of EpCAM and ITGA3 in the liver‐derived EVs are linearly associated with the hepatic expressions of EPCAM and ITGA3 (*r* = 0.9289 and *r* = 0.8703, *p* < 0.0001 and *p* < 0.0002, respectively, Figure 5C), we further investigated whether EpCAM and ITGA3 were continuously increasing through the development of MASLD by examining the hepatic expressions of EPCAM and ITGA3. Using a previously published hepatic transcriptomic dataset of 217 MASLD patients across all MASLD stages (Govaere et al. 2020), we found that the hepatic expressions of EPCAM and ITGA3 were continuously increased along disease progression (*r* = 0.8964 and *r* = 0.9193, respectively, Figure 5D). These results indicate that the EpCAM and ITGA3 in liver‐derived EVs were likely also increasingly associated with the disease progressions and might be potential biomarkers for MASLD.

## 4 Discussion

In this paper we are the first to isolate and characterize the EVs derived from human PCLS prepared from healthy and MASH livers. Our PCLS‐derived EVs showed good purity and the typical EV characteristics by western blots, proteomics and TEM analysis. Importantly, MASH EVs and healthy EVs exhibited great differences regarding their protein compositions, with around 300 proteins differently expressed. These significantly different proteins reflected the pathological changes of MASH and showed potential in promoting fibrosis. Functionally, MASH EVs promoted the activation of hepatic stellate cells, which coincides with previous reports (Eguchi et al. 2022; Kostallari et al. 2018). Furthermore, the protein levels of EpCAM and ITGA3 in liver‐derived EVs were positively correlated to the severity of hepatic fibrosis and were increasingly associated with the progression of MASLD. Therefore, EpCAM and ITGA3 in liver‐derived EVs could be potential biomarkers for MASLD.

As mentioned above, EVs isolated from PCLS of MASH patients, specifically their protein compositions, reflect the pathological changes of MASH. As shown in Figure 3D, the significantly up‐regulated proteins in MASH EVs are highly enriched in the ‘complement and coagulation cascade,’ which manifested the abnormally activated platelets and the trend toward the pro‐coagulant state of the patients with MASH (Ogresta et al. 2022; Kotronen et al. 2011). Meanwhile, the significantly down‐regulated proteins are highly abundant for modulating xenobiotics or drug metabolisms. Multiple pieces of evidence have shown that MASLD patients demonstrate altered levels of drug‐metabolizing enzymes, including both phase I and phase II enzymes (Merrell and Cherrington 2011), which may increase the risk for adverse drug reactions.

EVs are important intercellular and inter‐organ messengers that can transport biomolecules and regulate signalling pathways, thus facilitating intercellular and inter‐organ communication. EVs could directly interact with the target cells through their membrane‐bound ligands to the recipient cell receptors. EVs could also internalize into the recipient cells by fusion with either their plasma membrane or their endosomal membrane after endocytic uptake (Mathieu et al. 2019; Mulcahy et al. 2014). Both internalization routes would lead to the transfer of EV ligands and receptors to the recipient cells, which could subsequently alter the activation of the downstream signalling pathways. Several studies have reported that EVs derived from either parenchymal or non‐parenchymal cells in the liver could participate in the progression of MASLD, by promoting fibrosis via EV‐bounded proteins, for example, PDGFRα, VNN1, integrin B1 and CD147 (Guo et al. 2019; Kostallari et al. 2018; Kornek et al. 2011; Povero et al. 2013). In the present study, we observed that the CM of MASH PCLS significantly increased intracellular collagen type 1 and αSMA in LX2 cells (*p* < 0.05, Figure 1E), which indicated that MASH livers secreted pro‐fibrotic factors. Importantly, MASH EVs also significantly increased intracellular collagen type 1 and αSMA in LX2 cells (*p* < 0.01, Figure 4E), implying that EVs mediated the fibrogenic effects. From our proteomic result, we found that our MASH EVs were enriched with the previously reported EV‐bounded pro‐fibrotic proteins (PDGFRα, VNN1, integrin B1 and CD147). In addition, our MASH EVs were significantly enriched with several ligands that are crucial in mediating liver fibrosis, including PDGFB, LTBP1 and TIMP3. PDGFB could directly activate fibrogenesis via the PDGFB/PDGFRβ pathway. LTBP1 is a functional regulator of latent TGFβ1 that could activate latent TGFβ1, thereby stimulating the TGFβ signalling pathways. TIMP3 inhibits the degradation of extracellular matrix. These results showed that human PCLS‐derived EVs functionally represented the pathological roles of EVs in MASLD.

On the other hand, EVs demonstrated potential in the use as diagnostic and prognostic biomarkers. In patients with cirrhosis, hepatocyte‐derived EVs were increased in the plasma, and the induction of the EV population may assist in the prediction of mortality (Payance et al. 2018). In our current study, we also observed an increase in the number of MASH EVs compared to healthy EVs (*p* < 0.001, Figure 2D). Importantly, EV proteins EpCAM and ITGA3 were positively correlated to the secretion of pro‐collagen 1a1 and were associated with the disease progression across the whole disease spectrum (Figure 5). EpCAM and ITGA3 are both membrane‐bound proteins, which were suggested may serve as biomarkers for several types of carcinomas (Liao et al. 2022; Zhang et al. 2021; Jiao et al. 2019; Schulze et al. 2013; Nagata et al. 2013). Recently, a study showed that the EpCAM + CD133 + EVs isolated from the plasma are significantly increased in MASLD patients and animal models (Munoz‐Hernandez et al. 2023). It would be of great interest to validate the specificity and sensitivity of liver EVs associated with EpCAM and ITGA3 as biomarkers for MASLD. In addition, we compared our liver‐derived EVs to a previously published dataset of circulating EVs isolated from the serum of MASLD patients (Povero et al. 2020) with regard to their protein composition. As shown in Figure S6A, there were only 10% of proteins present in both liver‐derived EVs and circulating EVs. Moreover, among the significantly different proteins in MASH versus healthy, only 5 proteins demonstrated accordant increase (IGHA1, PLA2G2A and EPHA2) or decrease (PON1 and SERPINE1) in both liver‐derived EVs and circulating EVs (Figure S6B). This indicates a discrepancy of the EV populations isolated from serum or tissue. Therefore, it would be important to ascertain the tissue of origin of the circulating EVs and to assess whether liver‐derived EVs could be a better strategy for applying EVs as biomarkers in liver diseases.

We acknowledge that the current study has a limited sample size and lacks liver tissue for PCLS from the early stages of MASLD. Future validations are needed to verify the liver‐derived EV proteins as biomarkers in the early stages of the disease and to determine the cellular origin of these EVs. However, to our knowledge, there are currently limited reliable methods for accurately distinguishing the cellular origin of EVs. We attempted to computationally deconvolute our proteomic results from healthy and MASH cirrhotic EVs using recently published snRNA‐seq data from liver biopsies of healthy and MASH cirrhotic patients (Gribben et al. 2024). The deconvolution analysis indicated that, for healthy liver‐derived EVs, the majority of EVs originated from hepatocytes, with smaller contributions from macrophages, lymphocytes, and hepatic stellate cells. In contrast, for MASH cirrhotic liver‐derived EVs, most EVs were released from hepatocytes, lymphocytes, and hepatic stellate cells, with smaller contributions from cholangiocytes, macrophages, and neutrophils (Figure S7). It is important to note that these shifts in EV sources also reflect changes in the cellular composition of MASH cirrhotic livers compared to healthy livers. In addition, although there are a lot of significantly different proteins between MASH EVs and healthy EVs, we only observed a limited influence of the incubation media on EV protein composition (Figure 3C and Figure S4B), which might be due to the short incubation time of PCLS (24 h). Further experiments are needed to check whether prolonged incubation would alter the EV compositions.

In conclusion, as a ‘proof‐of‐concept,’ we are the first to isolate EVs from PCLS. EVs derived from MASH PCLS demonstrated the pathological characteristics of MASLD. The different protein cargos in EVs from MASH versus healthy PCLS may assist future studies in understanding the pathogenesis of MASLD and MASLD‐associated complications, as well as in applying EVs as biomarkers for MASLD.

## Author Contributions

**Yana Geng**: conceptualization (equal), data curation (lead), formal analysis (lead), investigation (lead), methodology (equal), writing – original draft (lead). **Ke Luo**: formal analysis (supporting), investigation (supporting), methodology (supporting) **Janine Stam**: methodology (equal), writing – review and editing (supporting). **Dorenda Oosterhuis**: investigation (supporting), methodology (supporting). **Alan R. Gorter**: investigation (supporting), methodology (supporting). **Marius van den Heuvel**: formal analysis (supporting). **Rossella Crescitelli**: formal analysis (supporting), writing – review and editing (equal). **Vincent E. de Meijer**: resources (equal), writing – review and editing (supporting). **Justina C. Wolters**: investigation (supporting), methodology (equal), writing – review and editing (equal). **Peter Olinga**: conceptualization (equal), project administration (lead), resources (equal), supervision (lead), writing – review and editing (equal).

## Ethics Statement

This study was approved by the Medical Ethical Committee of the University Medical Centre Groningen (UMCG), according to Dutch legislation and the Code of Conduct for dealing responsibly with human tissue in the context of health research (www.federa.org), forgoing the need for written consent for ‘further use’ of coded‐anonymous human tissue.

## Conflicts of Interest

Rossella Crescitelli has developed multiple EV‐associated patents for putative clinical utilization. Rossella Crescitelli owns equity in Exocure Bioscience Inc.

## Supporting information



Supporting Information

Supporting Information

Supporting Information

Supporting Information

## Data Availability

The MS‐based proteomics data used in this study is available upon request from the corresponding author.
