# Supplementary material for: Characterization of Extracellular Vesicles Derived From Human Precision‐Cut Liver Slices in Metabolic Dysfunction‐Associated Steatotic Liver Disease
Source: J Extracell Biol. 2025 Apr 30;4(5):e70043. doi: 10.1002/jex2.70043 (PMC12042696; doi:10.1002/jex2.70043)
Supplement: Supplementary file 1 — Supporting Information [file JEX2-4-e70043-s001.docx]

Characterization of extracellular vesicles derived from human precision-cut liver slices in metabolic dysfunction-associated steatotic liver disease

Yana Geng ^1^, Ke Luo ^1^, Janine Stam ^1, 2^, Dorenda Oosterhuis ^1^, Alan R. Gorter ^1^, Marius van den Heuvel ^3^, Rossella Crescitelli ^4^, Vincent E. de Meijer ^5^, Justina C. Wolters ^6^, Peter Olinga ^1,*^

**Supplementary materials:**

**
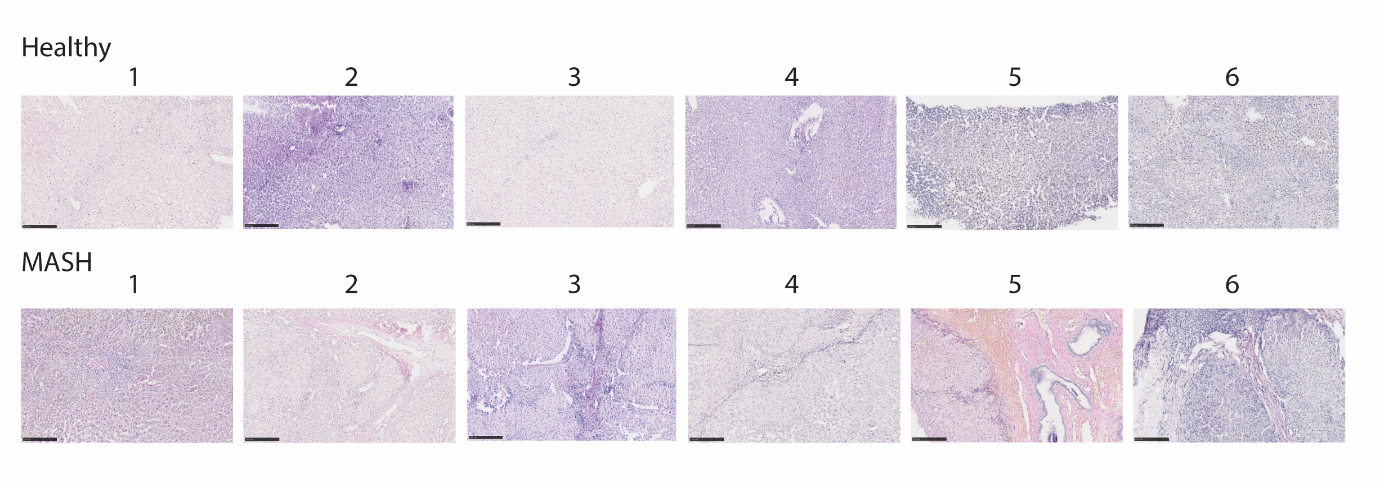
**

Supplementary Figure 1. The H&E staining images of the livers used in this study.

**
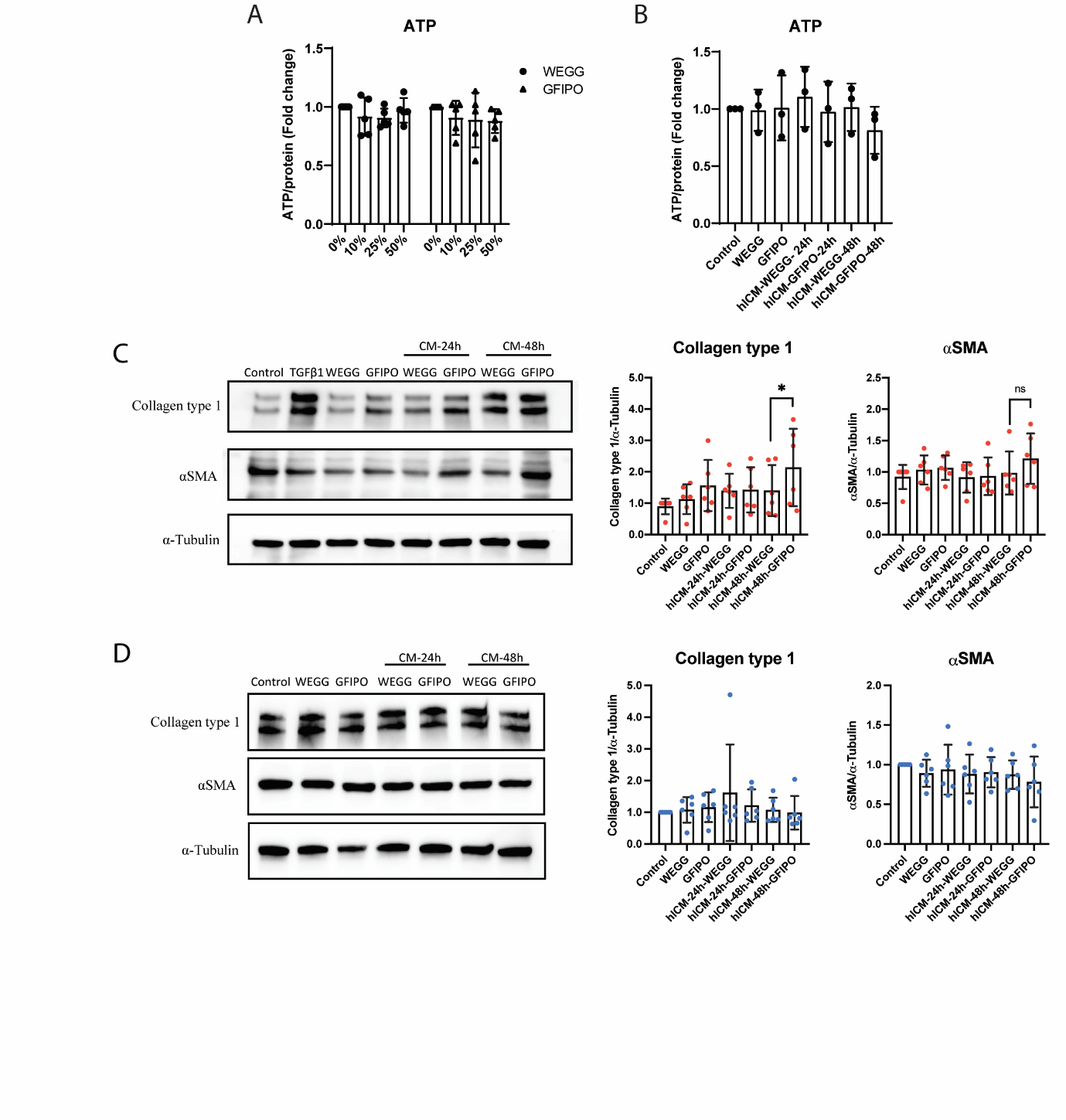
**

Supplementary Figure 2. (A) LX2 cells were incubated in different percentages of slice medium (WEGG or GFIPO) for 48 hours. ATP levels were measured and normalized to the 0% groups. n = 5. (B) LX2 cells were incubated in 25% (v/v) of slice medium or conditioned medium (CM) collected from healthy PCLS at 24 or 48 hours. After 48 hours, cells were collected and intracellular ATP was measured. n = 3.

*
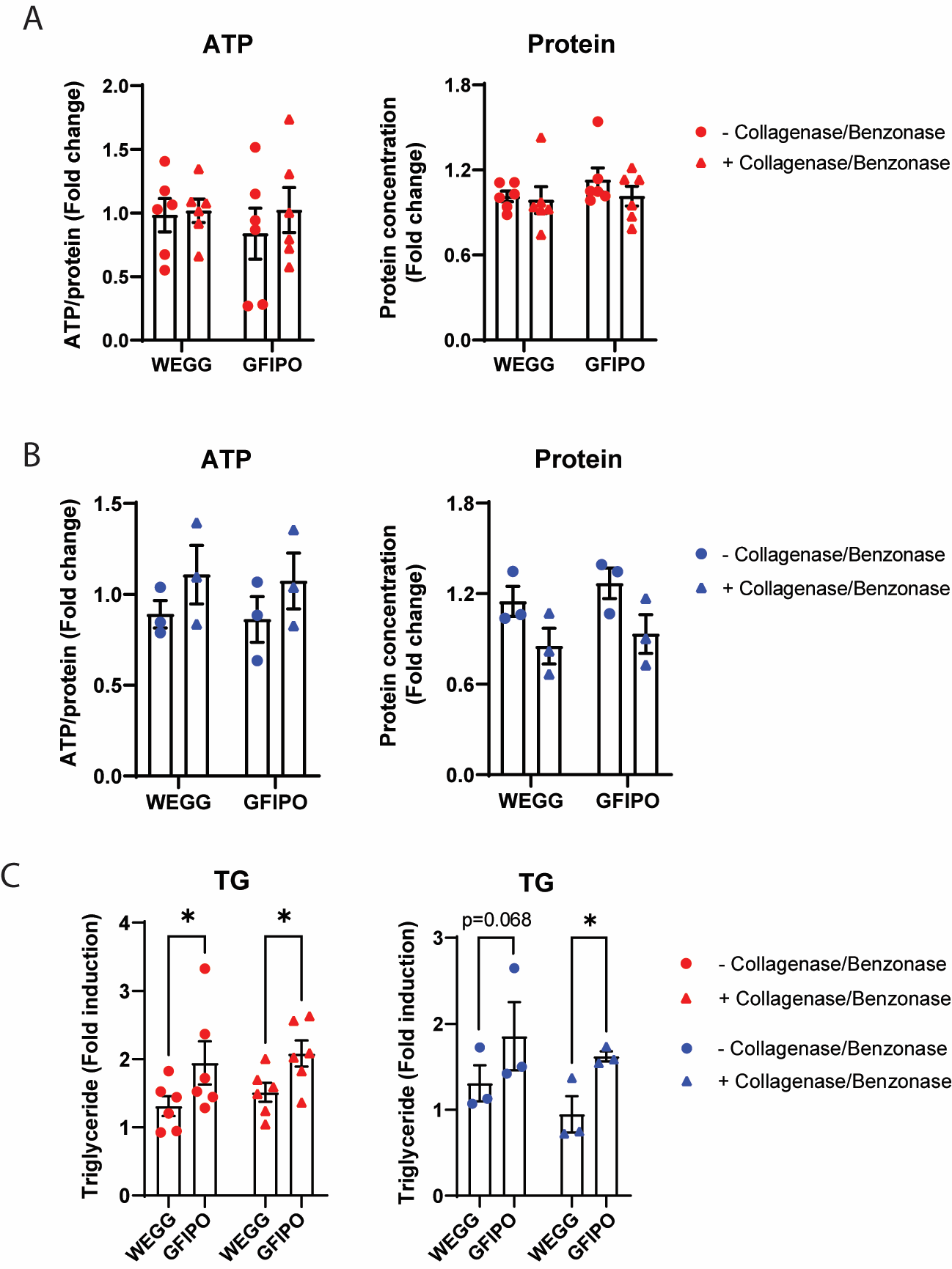
*

Supplementary Figure 3. (A) Healthy and (B) MASH PCLS were incubated for 48 hours with or without collagenase (2 mg/ml) and benzonase (50 U/ml) during the last 24 hours. ATP and Protein amounts were measured. (C) Triglyceride levels of healthy (red) and MASH (blue) PCLS incubated with or without collagenase and benzonase. * indicates p < 0.05.

*
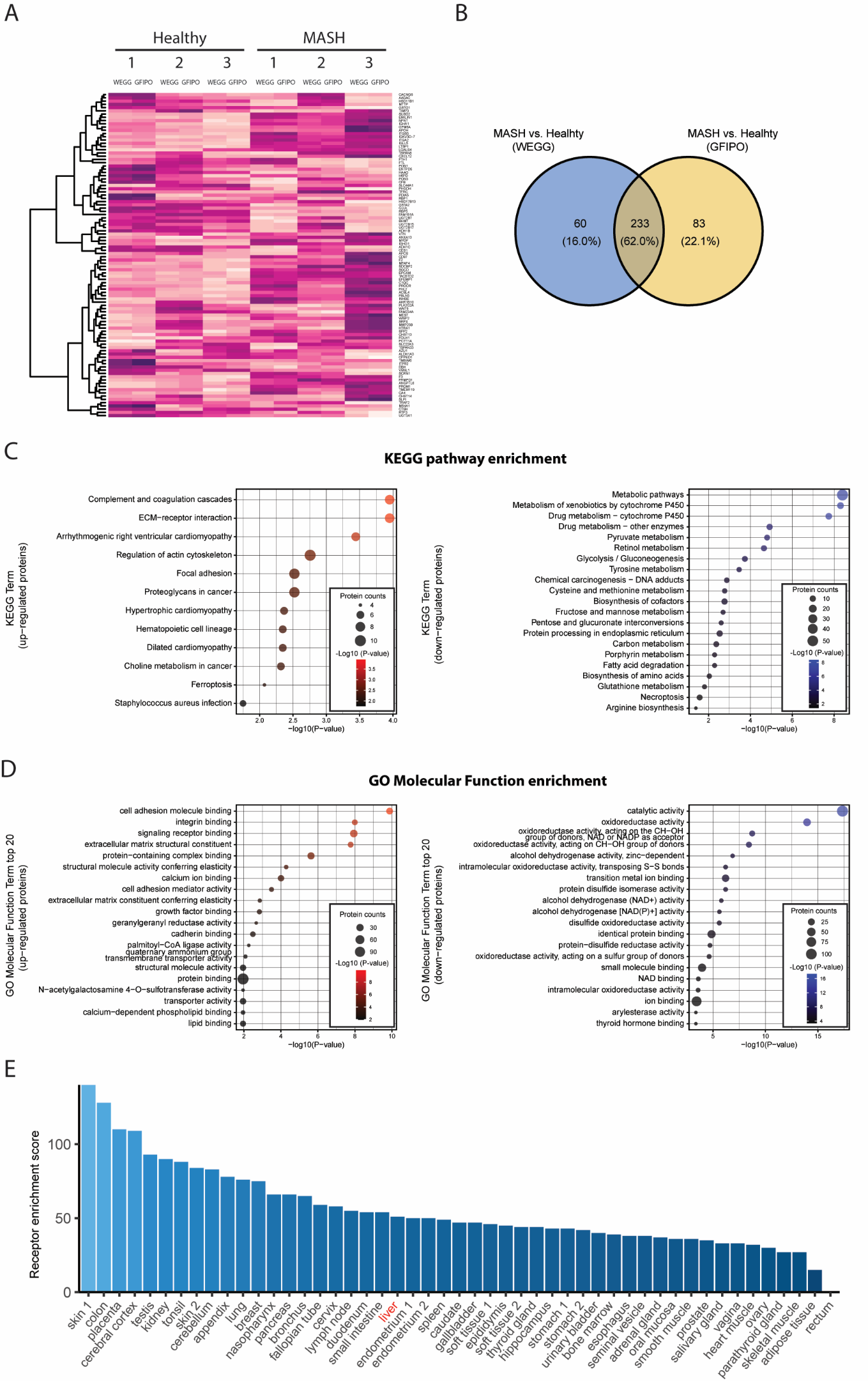
*

Supplementary Figure 4. (A) Heatmap of the top 100 significantly different proteins between EVs derived from MASH PCLS and EVs derived from healthy PCLS among all EV samples. (B) Venn diagram of the significantly different proteins in EVs from MASH WEGG vs healthy WEGG and the significantly different proteins in EVs from MASH GFIPO vs healthy GFIPO. (C) KEGG pathway enrichment analysis of the significantly up- or down-regulated EV proteins in MASH (GFIPO) EVs versus healthy (GFIPO) EVs. (D) GO molecular function enrichment analysis of the significantly up- or down-regulated EV proteins in MASH (GFIPO) EVs versus healthy (GFIPO) EVs. (E) Bar plots represent tissue enrichments of EV protein associated receptors based on the data from The Human Protein Atlas, https://www.proteinatlas.org/.

Supplementary Figure
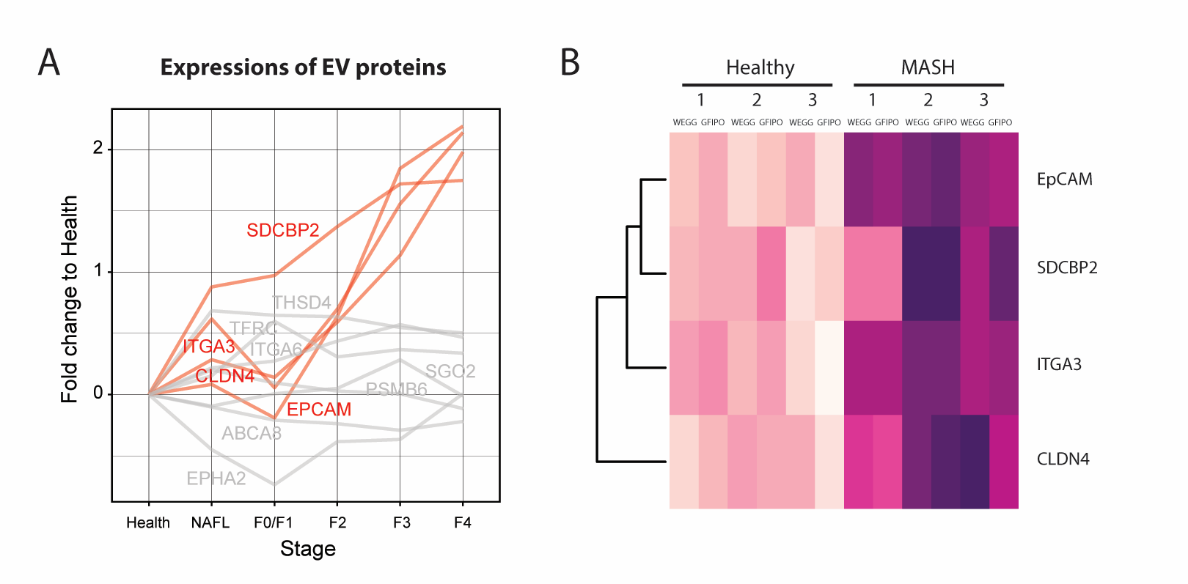
5. (A) The expressions of EV-enriched proteins in the liver across MASLD stages [37]. (B) A heatmap of the protein levels of EpCAM, SDCBP2, ITGA3 and CLDN4 in all EV samples.


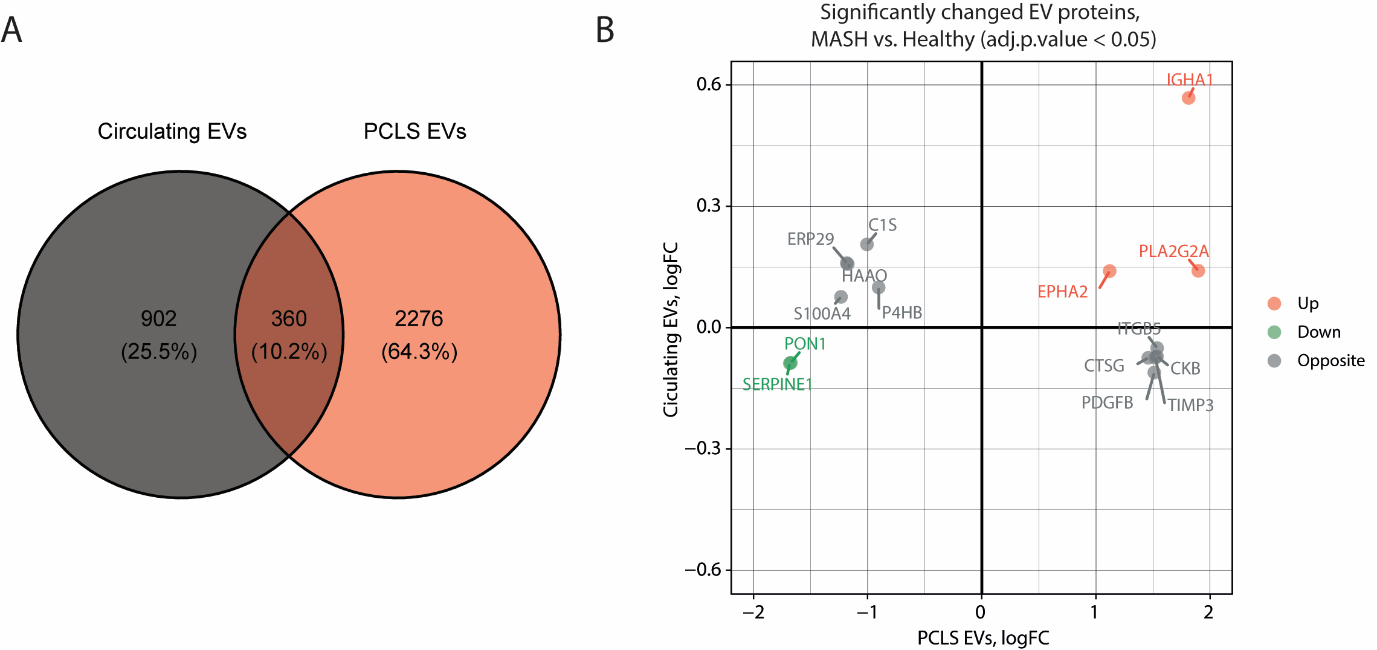


Supplementary Figure 6. Comparison of EV proteins between PCLS EVs and circulating EVs. (A) Venn diagram of the proteins in circulating EVs isolated from serum [1] and PCLS EVs. (B) The significantly changed proteins in both PCLS EVs and circulating EVs.


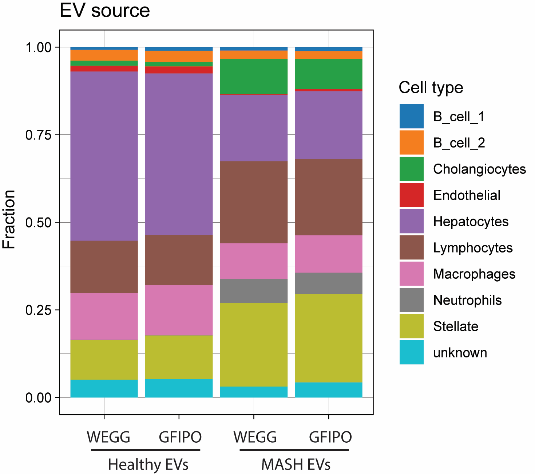


Supplementary Figure 7: proteomic results of healthy and MASH cirrhotic EVs were deconvoluted using recently published snRNA-seq data from liver biopsies of healthy and MASH cirrhotic patients [2] by BayesPrism.

Supplementary Table 1. List of primary antibodies

| Protein | Clonality | Dilution | Source |
| --- | --- | --- | --- |
| Collagen type 1 | Polyclonal | 1:2000 | SouthernBiotech |
| α-SMA | Monoclonal | 1:2000 | Sigma-Aldrich |
| α-tubulin | Monoclonal | 1:1000 | Sigma-Aldrich |
| CD9 | Monoclonal | 1:500 | Millipore |
| CD81 | Monoclonal | 1:500 | Abcam |
| Rab7 | Monoclonal | 1:1000 | Cell Signaling Technology |
| Cyt-c | Monoclonal | 1:1000 | Cell Signaling Technology |
| Calnexin | Monoclonal | 1:2000 | BD Biosciences |

Supplementary Table 2. Histological scores of the liver biopsies

| Liver number | Group | Histological scores | | | | Proteomics |
| --- | --- | --- | --- | --- | --- | --- |
|  |  | Steatosis | Hepatocyte ballooning | Lobular inflammation | Fibrosis |  |
| HL1 | Healthy | 0 | 0 | 0 | 0 |  |
| HL2 |  | 0 | 0 | 0 | 0 |  |
| HL3 |  | 0 | 0 | 0 | 0 |  |
| HL4 |  | 0 | 0 | 0 | 0 | √ |
| HL5 |  | 0 | 0 | 0 | 0 | √ |
| HL6 |  | 0 | 0 | 0 | 0 | √ |
| NL1 | MASH | 0 | 0 | 0 | 3 |  |
| NL2 |  | 0 | 0 | 2 | 4 | √ |
| NL3 |  | 0 | 0 | 0 | 3 |  |
| NL4 |  | 0 | 0 | 0 | 4 |  |
| NL5 |  | 0 | 0 | 1 | 4 | √ |
| NL6 |  | 0 | 1 | 2 | 4 | √ |

**Reference:**

1. Povero, D., et al., *Characterization and Proteome of Circulating Extracellular Vesicles as Potential Biomarkers for NASH.* Hepatol Commun, 2020. **4**(9): p. 1263-1278.

2. Gribben, C., et al., *Acquisition of epithelial plasticity in human chronic liver disease.* Nature, 2024. **630**(8015): p. 166-173.
